# Effects of Differential Shading on Summer Tea Quality and Tea Garden Microenvironment

**DOI:** 10.3390/plants13020202

**Published:** 2024-01-11

**Authors:** Shibei Ge, Yameng Wang, Keyin Shen, Qianying Wang, Golam Jalal Ahammed, Wenyan Han, Zhifeng Jin, Xin Li, Yuanzhi Shi

**Affiliations:** 1 Tea Research Institute, Chinese Academy of Agricultural Science, Hangzhou 310008, China; geshibei@tricaas.com (S.G.); hanwy@tricaas.com (W.H.); 2 College of Horticulture and Plant Protection, Henan University of Science and Technology, Luoyang 471023, China; ahammed@haust.edu.cn; 3 Zhejiang Climate Center, Hangzhou 310056, China; jzfeng0423@163.com

**Keywords:** tea plants (*Camellia sinensis* L.), shading, tea quality, tea polyphenol, amino acids, microenvironment, far-red light

## Abstract

Shading is an effective agronomic technique to protect tea plants from intense sunlight. However, there are currently very few studies on more effective shading methods to improve the quality of summer tea. In this study, ‘Longjing43’ plants were grown under four different shading treatments for 14 days, with no shading as the control. Among the four shading treatments, double-layer-net shadings had the most positive impact on the tea quality, resulting in higher levels of amino acids but lower levels of tea polyphenols. Additionally, double-layer-net shadings provided more suitable microenvironments for tea plants. The tea leaves in T4 (double nets 50 cm above the plant canopy) contained 16.13 mg∙g^−1^ of umami and sweet amino acids, which was significantly higher than in other treatments. T4 had the lowest air temperature and the most suitable and stable soil water content. Interestingly, the ratio of red light to far-red light in T4 was only 1.65, much lower than other treatments, which warrants further study. In conclusion, the microenvironment induced by shading can greatly affect the tea quality, and double-layer-net shading is better for improving the quality of summer tea.

## 1. Introduction

Tea is a widely consumed non-alcoholic beverage made from the leaves of tea plants (*Camellia sinensis* L.). It has numerous health benefits, such as improving sleep quality, protecting neurons, enhancing attention, and promoting anti-oxidative, anti-inflammatory, anti-tumor, and cardioprotective effects [1,2]. These health benefits are attributed to its three main functional compounds, namely, tea polyphenols, amino acids, and caffeine, which account for 15–30%, 1–3%, and 2–4% of the dry weight of tea leaves, respectively [3,4,5]. Therefore, tea polyphenols, amino acids, and caffeine are the three main quality components of tea. High levels of tea polyphenols and caffeine in tea are responsible for its bitter and astringent taste, while amino acids, especially theanine, are associated with its sweetness and umami taste [6]. The quality of tea buds determines the economic value of tea. Nonetheless, the quality of fresh tea leaves is influenced by the harvest season [7,8]. In China, tea produced in the spring is considered the best among the four seasons. Although summer and autumn tea accounts for more than 50% of the annual output, this tea is of poor quality and largely discarded. Therefore, it is of great significance to improve the quality of summer tea by developing effective agronomic techniques for maximizing the economic value of tea gardens.

Shading is a conventional agronomic technique to protect tea trees from intense sunlight and high temperatures in the summer. Studies have found that shading could enhance the accumulation of chlorophyll and quality compounds [9,10]. Therefore, improving the shading technique is crucial to increasing the utilization of summer tea. A previous study compared the effects of different shading times and degrees on the metabolites of fresh tea leaves and found that the tea quality was positively related to the duration and degree of shading [11]. Shading-modulated light and temperature, two important environmental factors, could potentially improve the quality of tea by affecting the synthesis and accumulation of vital tea metabolites [9,12]. However, there are fewer studies on the improvement of shading methods, and the main microenvironmental factors affecting the quality of fresh tea leaves under shading remain elusive.

To investigate an improved shading technique, this study used *Camellia sinensis* cv. Longjing43 plants and set up four different shading treatments, considering the number of shading nets and the distance from the shading nets to the canopy of tea trees. The objective of the present study was to reveal the microenvironmental factors that influence the quality of tea leaves and advocate a more efficient shading method for improving the quality of summer tea.

## 2. Results

### 2.1. Photosynthesis and Tea Yield as Influenced by Different Shading Treatments

In this study, ‘Longjing43’ plants were grown under five treatments, respectively. T1 was one shading net on the canopy of tea plants; T2 was double shading nets on the canopy of tea plants; T3 was one shading net 50 cm above the canopy of tea plants; T4 was double shading nets 50 cm above the canopy of tea plants; and the control was no shading.

**Figure 1:**
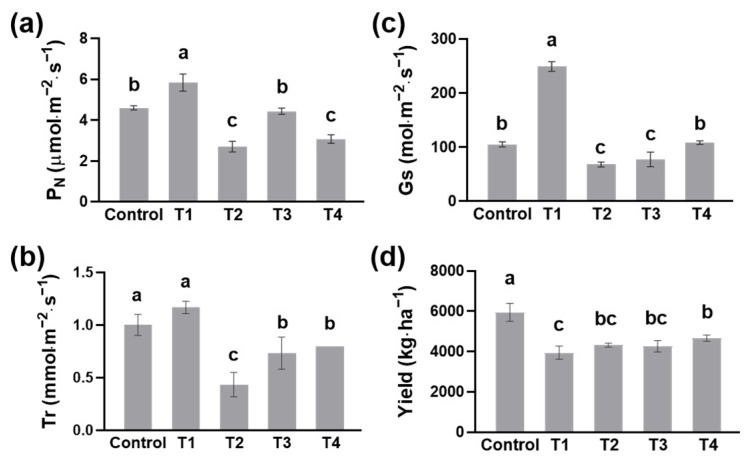
Photosynthesis and tea yield as influenced by different shading treatments. (**a**) Net photosynthetic rate (P_N_), (**b**) transpiration rate (Tr), (**c**) stomatal conductance (Gs), and (**d**) the yield of fresh tea leaves. Fifteen-year-old ‘Longjing 43’ tea plants were nurtured at four shading treatments and no shading treatment (control). After 14 days of treatment, the photosynthesis parameters were determined on the first and second mature leaves below one bud and two leaves. For measuring the yield of tea, one bud and two leaves were collected. Data are presented as the average of three biological replicates (±SD). Shared letters indicate no statistically significant difference in the means (*p* > 0.05) according to the *LSD* test.

To understand the response of tea plants to different shading treatments, we tested the photosynthetic characteristics of tea plants on the morning of the 14th day and then measured the yield of tea. The results showed that the net photosynthetic rate (P_N_) of T2 and T4 tea plants was lower than that of control tea plants, and the P_N_ of T2 was only half that of the control (Figure 1a). Similarly, the transpiration rate (Tr) and stomatal conductance (Gs) of T2 tea plants were the lowest among the five treatments (Figure 1b,c). Among the four shading treatments, tea plants in T1 showed the highest promotion in P_N_, Tr, and Gs (Figure 1a). The yield of fresh tea leaves in the control was 5944 kg·ha^−1^, while the yield in shading treatments decreased significantly (Figure 1d). There was no significant difference in tea yield among T2, T3, and T4 treatments (Figure 1d). However, T1 had the lowest yield of fresh tea leaves (3939 kg·ha^−1^), only 66% of that in the control (Figure 1d). These results indicated that shading treatments, especially T2 and T4, inhibited tea growth by reducing the photosynthesis of tea plants.

### 2.2. Nutrient Elements in Tea Leaves as Influenced by Different Shading

**Figure 2:**
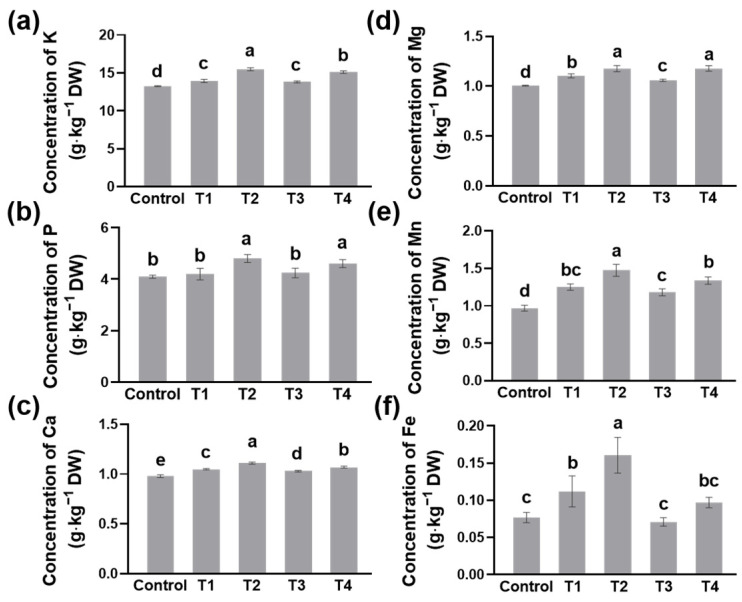
The concentration of nutrient elements in tea leaves as influenced by different shading treatments. The concentrations of (**a**) potassium (K), (**b**) phosphorus (P), (**c**) calcium (Ca), (**d**) magnesium (Mg), (**e**) iron (Fe), and (**f**) manganese (Mn) in tea leaves. Fifteen-year-old ‘Longjing 43’ tea plants were nurtured at four shading treatments and no shading treatment (control). After 14 days of treatment, one bud and two leaves of tea plants were sampled to measure the concentration of nutrient elements. Data are presented as the average of three biological replicates (±SD). Shared letters indicate no statistically significant difference in the means (*p* > 0.05) according to the *LSD* test.

To study tea quality under different shading treatments, we tested the concentrations of nutrient elements in tea leaves. Shading treatments in most cases increased the concentrations of macronutrients such as potassium (K), phosphorus (P), calcium (Ca), and magnesium (Mg), as well as microelements like iron (Fe) and manganese (Mn) (Figure 2a–f). Among the four shading treatments, T2 was the most efficient treatment to promote nutrient absorption, followed by T4 (Figure 2a–f). In T2, the concentration of K in tea leaves was about 15.5 g∙kg^−1^, while that in the control was only 13.3 g∙kg^−1^ (Figure 2a). At the same time, the concentrations of Mn and Fe were almost 1.5 and 2 times higher than the control, respectively (Figure 2e,f). Compared to the control, T3 did not affect the accumulation of P and Fe (Figure 2b,f). These results indicated that T2 and T4 treatments were beneficial to the nutrient accumulation of summer tea.

### 2.3. The Quality of Tea as Influenced by Different Shading

**Figure 3:**
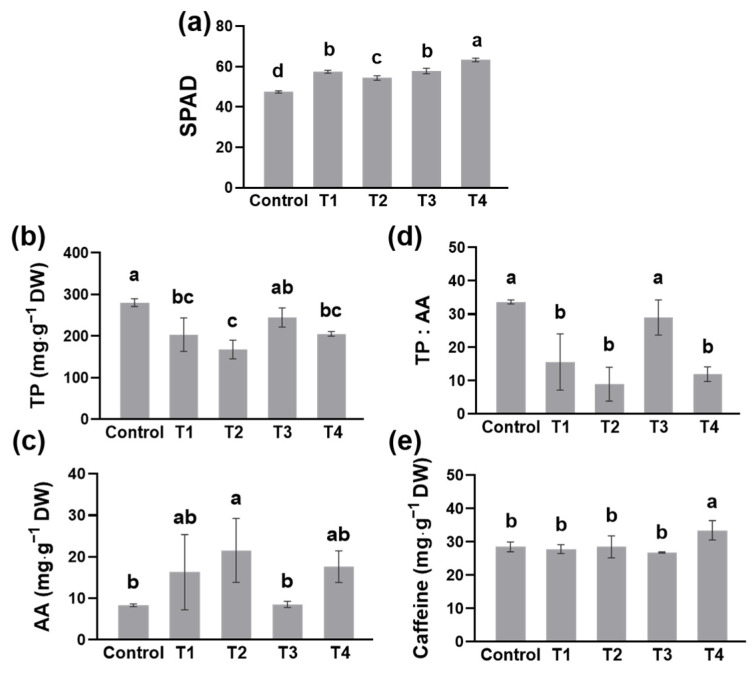
SPAD and quality components in tea leaves as influenced by different shading treatments. (**a**) SPAD, (**b**) the concentration of tea polyphenols (TP), (**c**) the concentration of total amino acids (AA), (**d**) the ratio of tea polyphenols and total amino acids (TP:AA), and (**e**) the concentration of caffeine. Fifteen-year-old ‘Longjing 43’ tea plants were nurtured at four shading treatments and no shading treatment (control). After 14 days of treatment, SPAD values were determined on the first and second mature leaves below one bud and two leaves. One bud and two leaves of tea plants were sampled to measure the tea quality. Data are presented as the average of three biological replicates (±SD). Shared letters indicate no statistically significant difference in the means (*p* > 0.05) according to the *LSD* test.

Next, we determined the quality of the fresh tea leaves. Leaf color is an important visual indicator of the quality of green tea. SPAD is the relative value of chlorophyll content, reflecting plant greenness. After 14 days of treatment, the SPAD values in the shading treatments increased when compared to the control (Figure 3a). Among the four shading treatments, T4 had the most significant effect on the accumulation of chlorophylls in tea leaves, followed by T3, T1, and T2. Tea polyphenols (TP), amino acids (AA), and caffeine are the three main quality compounds in tea. Under the condition of no shading, the TP content in tea leaves was approximately 280 mg∙g^−1^ (Figure 3a). Except for T3, all shading treatments resulted in a reduction of TP content when compared to the control (Figure 3a). T2 significantly contributed to the decrease in TP content, accounting for 60% of the control (Figure 3a). On the contrary, the free AA content of tea in shading treatments was higher than that in the control, indicating that shading promoted free AA accumulation (Figure 3b). Among them, the free AA in T2 accounted for approximately 21.5 mg∙g^−1^, which was 2.5 times that in the control (Figure 3b). Therefore, except for T3, shading treatments decreased the ratio of TP to AA, meaning the quality of tea was improved by shading (Figure 3c). In addition, except for T4, shading did not affect the accumulation of caffeine in tea (Figure 3d).

**Figure 4:**
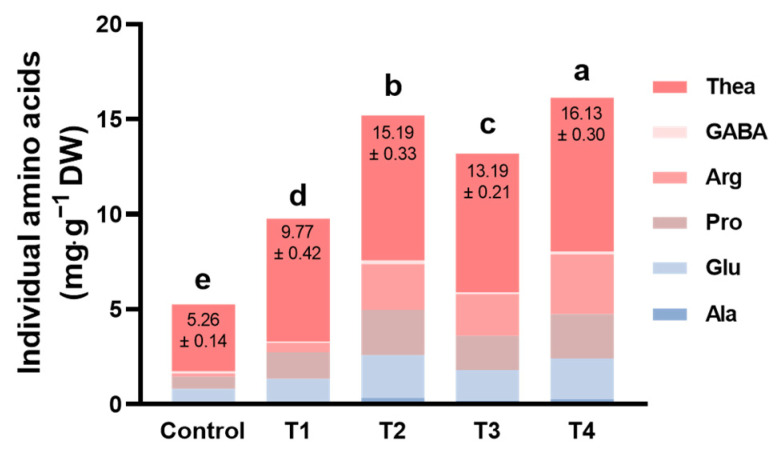
Individual amino acids in tea leaves as influenced by different shading treatments. Fifteen-year-old ‘Longjing 43’ tea plants were nurtured at four shading treatments and no shading treatment (control). After 14 days of treatment, one bud and two leaves of tea plants were sampled to measure the concentrations of individual amino acids. Data are presented as the average of three biological replicates (±SD). Shared letters indicate no statistically significant difference in the means (*p* > 0.05) according to the *LSD* test.

A former study showed that approximately 70% of the umami taste of green tea is associated with amino acids, especially L-glutamic acid and its metabolic products [13,14]. As Figure 4 shows, theanine was the main amino acid of tea in all treatments. Compared to the control, shading significantly increased the contents of the tricarboxylic acid cycle metabolites, such as alanine (Ala), glutamate (Glu), proline (Pro), arginine (Arg), Gamma butyric acid (GABA), and theanine (Thea). Meanwhile, these amino acids of tea leaves in T4 were significantly accumulated among the four shading treatments (Figure 4). To sum up, shading treatments, especially T2 and T4, were helpful in accumulating more umami and sweet taste metabolites in tea leaves.

### 2.4. Temperature and Soil Water Content in the Tea Garden as Influenced by Different Shading

**Figure 5:**
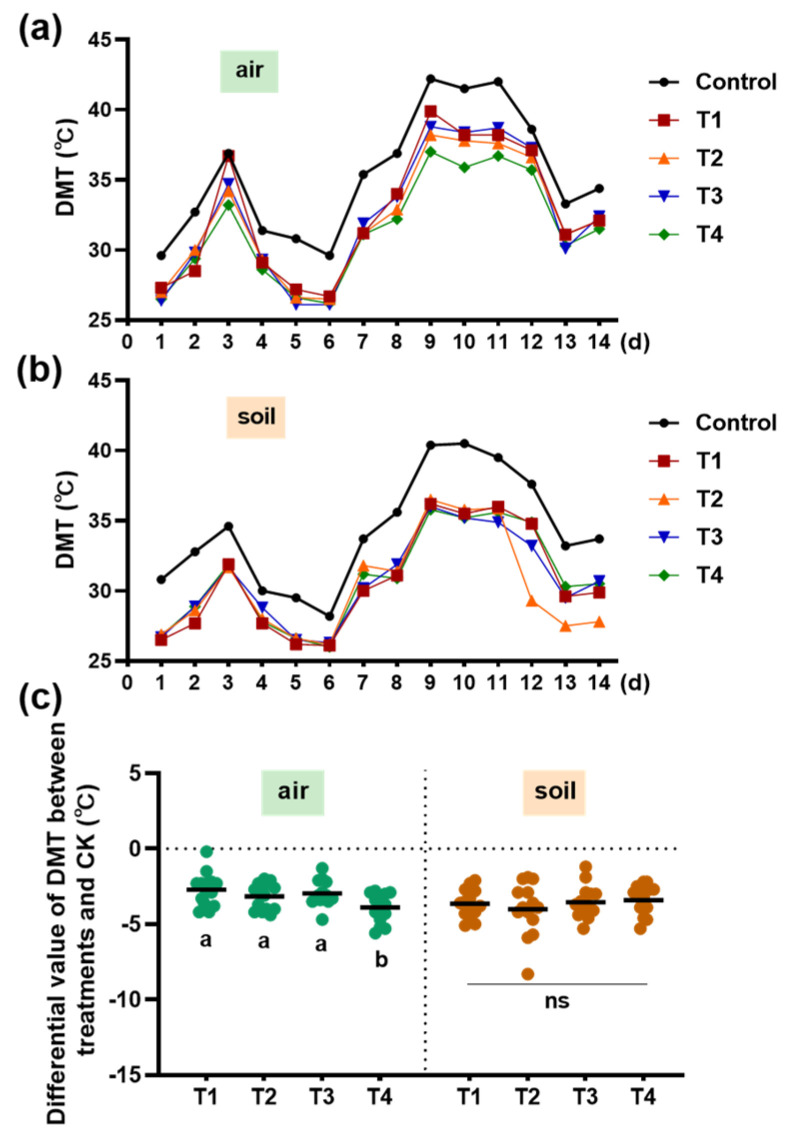
Temperature on the tea canopy and in the soil as influenced by different shading treatments. (**a**) Daily maximum temperature (DMT) of the air on the tea canopy; (**b**) DMT in the soil; and (**c**) the differential value of DMT between the treatment and the control. Fifteen-year-old ‘Longjing 43’ tea plants were nurtured at four shading treatments and no shading treatment (control). Data in (**a**,**b**) are presented from 27 June 2021 to 10 July 2021. Data in (**c**) are presented as the average of fourteen differential values (±SD) from the fourteen days of treatment. Shared letters and ‘ns’ indicate no statistically significant difference in the means (*p* > 0.05) according to the *LSD* test.

Temperature and soil water content are two main environmental factors that can be manipulated by shading to affect the growth of tea plants. Here, we monitored the temperature on the canopy of tea plants and in the soil during the whole experiment. As shown in the control, there were 7 days of daily maximum temperatures (DMTs) above 35 °C both in air and in soil (Figure 5a,b). Although the change trends of DMT in the shading treatments were similar to those in the control, the DMTs were much lower than those in the control (Figure 5a,b). Then, we calculated the differential value of DMT between the shading treatments and the control. Among the four shading treatments, T4 had a great differential value of DMT in air (Figure 5c). However, there was no difference between the four shading treatments in the differential DMT value in soil (Figure 5c).

**Figure 6:**
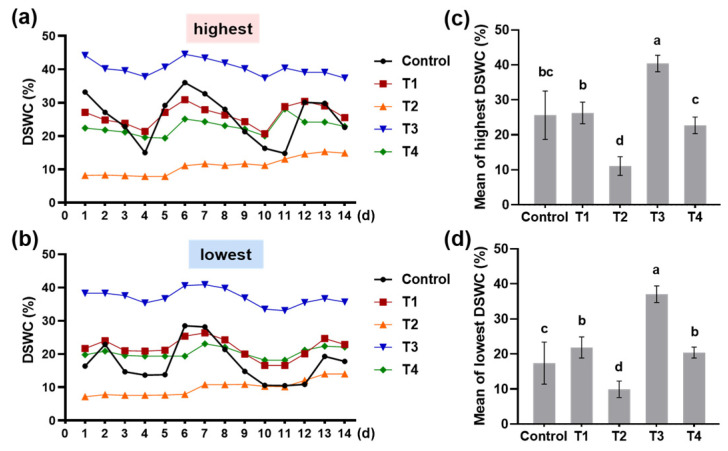
Soil water content of the tea garden as influenced by different shading treatments. (**a**) The highest value of daily soil water content (DSWC); (**b**) the lowest value of DSWC; (**c**) the average of the highest value of DSWC; and (**d**) the mean of the lowest value of DSWC. Fifteen-year-old ‘Longjing 43’ tea plants were nurtured at four shading treatments and no shading treatment (control). Data in (**a**,**b**) are presented from 27 June to 10 July 2021. Data in (**c**,**d**) are presented as the average of fourteen values (±SD) from the fourteen days of treatment. Shared letters indicate no statistically significant difference in the means (*p* > 0.05) according to the *LSD* test.

Soil water content is a key environmental indicator of the tea garden. We recorded the daily soil water content (DSWC), compared the highest and lowest DSWC, and calculated the average DSWC. The DSWC in the control fluctuated dramatically during the 14 days of the experiment (Figure 6a,b). In contrast, shading improved the stability of the DSWC (Figure 6a,b). Meanwhile, the DSWC in T2 was much lower than that in the control, but the DSWC in T3 was much higher (Figure 6a–c). These results indicated that shading affected the temperature and soil water content in the tea garden.

### 2.5. Light Environment in the Canopy of Tea Plants as Influenced by Different Shading

The light environment is a vital part of the microenvironment in the tea garden. PPFD (photosynthetic photon flux density) on the canopy of tea plants was measured by PLA-30 at 10 o’clock every morning. As shown in Table 1, the PPFD varied considerably over 7 days. The average light transmittance (%) was determined based on the PPFD values (Table 1). Comparing the four shading treatments, single-layer-net shadings (T1 and T3) reduced about 90% of irradiance, while double-layer-net shadings (T2 and T4) cut the irradiance down by approximately 99% (Table 1).

**Figure 7:**
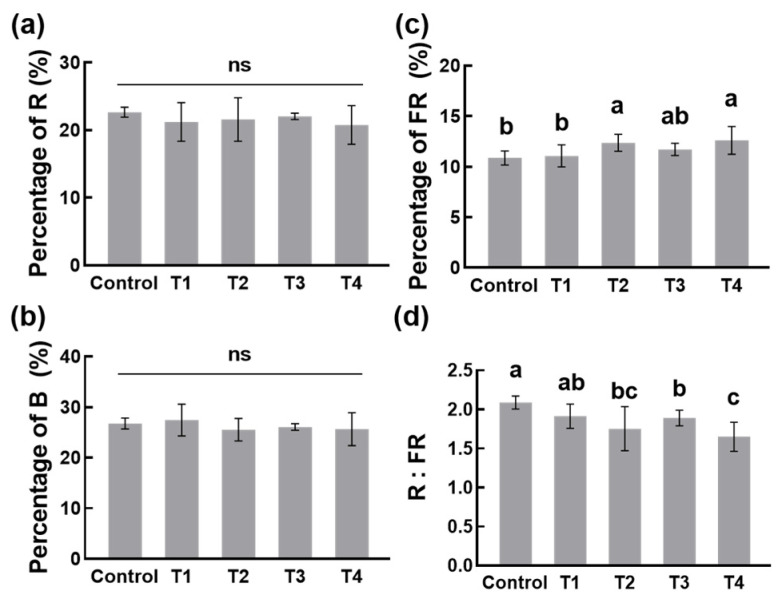
Light quality on the canopy of tea plants as influenced by different shading treatments. The percentage of (**a**) red (R, 600–700 nm) light, (**b**) blue (B, 400–500 nm) light, and (**c**) far-red (FR, 700–800 nm) light. (**d**) The ratio of R light to FR light (R:FR). Data are presented as the average of seven replicates (±SD) from the later seven days of treatment, from 4–10 July 2021. Shared letters and ‘ns’ indicate no statistically significant difference in the means (*p* > 0.05) according to the *LSD* test.

The total photometric irradiance and specific photometric irradiance were also measured by PLA-30. We analyzed the percentage of blue (B, 400–500 nm), red (R, 600–700 nm), and far-red (FR, 700–800 nm) light on the canopy of plants over the last 7 days of the experiment. The results showed that there were no differences in the percentage of R and B between the shading treatments and the control, respectively (Figure 7a,b). However, shading changed the percentage of FR, which was about 9.96% in the control, 11.55% in T2, and 11.66% in T4 (Figure 7c). The ratio of R to FR is a key indicator of the light environment, which decreased in shading treatments, and the reduction was significant in T2, T3, and T4 (Figure 7d). In addition, there were great differences among the four shading treatments, and the influence of T2 and T4 with double-layer shading nets on FR was greater than that of T1 and T3 using a single-layer shading net (Figure 7c,d). In summary, shading changed the light environment of the tea garden, including the light intensity and quality, especially FR.

## 3. Discussion

**Figure 8:**
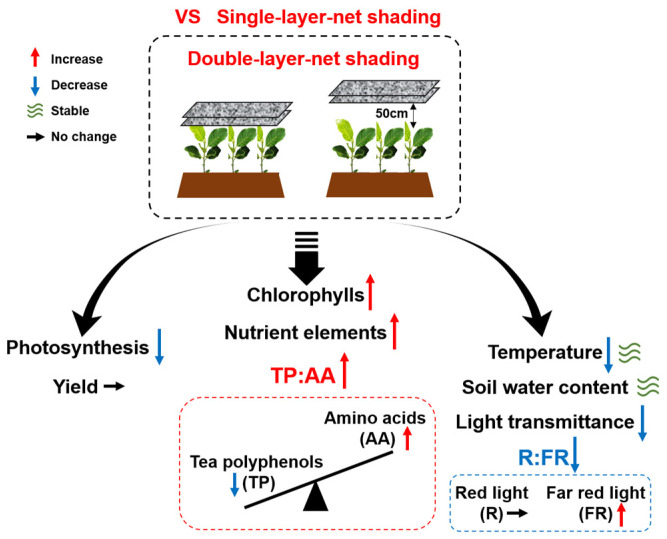
The impacts of double-layer-net shading on tea yield, quality, and microenvironment when compared with single-layer-net shading.

In the present study, we set up four different shading treatments to study plant photosynthesis, the yield of fresh tea leaves, nutrient contents, and quality components of tea leaves, as well as the microenvironmental factors of tea gardens. We found that shading reduced the yield of tea but improved the quality of tea leaves in summer (Figure 1d, Figure 2, Figure 3 and Figure 4). The results are consistent with a previous study that reveals that shading treatment improves the nutritional and sensory quality of green tea (Figure 2, Figure 3 and Figure 4) [15]. Therefore, the positive effect of shading on tea quality can be optimally utilized in tea cultivation and production in the summer. Here, we discussed the influence of shading methods on the yield and quality of tea, as well as the possible regulatory factors (Figure 8).

Shading greatly affected the photosynthesis of tea trees, in that the double-layer-net shadings significantly decreased the P_N_ (Figure 1a). In contrast, T1 with a single-layer shading net increased the P_N_, which may provide a mild condition for eliminating the photoinhibition of tea trees under excessive illumination (Figure 1a). Many factors affect tea production, with photosynthesis being the decisive factor. A previous study found that the yield of summer tea increased with the shading level after 9 days of shading while decreasing with the shading level after 14 days of shading [16]. Here, after 14 days of shading, there was no significant difference in the yields of summer tea among the four shading treatments (Figure 1b).

Contrary to tea yield, the quality of summer tea varies greatly with the different shading treatments. Double-layer-net shading treatments significantly improved the concentrations of nutrient elements in tea leaves (Figure 2a–f). Due to the fact that nutrients make up most of tea’s metabolites, these results predicted that shading was helpful in improving the quality of tea. When growing under shading, the leaf color of chlorotic tea cultivars such as ‘Huangjinya’, ‘Yu-Jin-Xiang’, and ‘Baijiguan’ turned green [17,18,19,20]. These natural phenomena directly show that shading or low light contributes to chlorophyll biosynthesis. As SPAD is relative chlorophylls, the results indicated that not only the shading level but also the height of shading nets affected the accumulation of chlorophylls (Figure 3a). Sano et al. [16] also revealed that the increasing shading level was beneficial for the accumulation of chlorophylls, resulting in higher SPAD values. In addition, Chen et al. [10] found that extending the shading time was positive for chlorophyll biosynthesis, and shading might promote the expression of *CsPORL-2* by inhibiting the expression of *CsHY5*, leading to a high accumulation of chlorophyll in tea leaves.

Previous studies found that long-term shading, about 16 days, promoted nitrogen metabolism but inhibited carbon metabolism, which had a good impact on the quality of tea [21]. However, Li et al. [22] inferred that the nitrogen metabolism in the leaves was promoted by short-term shading while inhibited by long-term shading. Here, we detected the quality of tea after 14 days of shading and found shading contributed to a better quality of summer tea (Figure 3b–d). T2 and T4 with a higher shading rate decreased the content of tea polyphenols and promoted the accumulation of free amino acids (Figure 3b,c). Therefore, T2 and T4 had a lower ratio of tea polyphenols to amino acids, resulting in better tea quality (Figure 3d). This result is similar to the conclusion of Ji et al. [11], that the contents of most amino acids were conspicuously elevated when shade levels were raised from 90% to 100%. Liao et al. [23] found that the height of shading nets affected the content of amino acids, which was not significantly different in our experiment (Figure 3c). Although some studies have found that shading could increase the content of caffeine in tea leaves, there were no differences between most shading treatments and the control (Figure 3e). Different from T2, both with two layers of shading nets, T4 elevated the content of caffeine in tea leaves (Figure 3e). Moreover, double-layer-net shadings accumulated more umami and sweet amino acids, especially theanine, than single-layer-net shadings (Figure 4). Up to now, more and more studies have confirmed that shading contributes to the accumulation of theanine, and the mechanism of theanine biosynthesis is promoted by shading [24,25]. This indicated that the double-layer-net shadings were good for the quality of tea leaves, and the shading height may also affect it.

Hot weather inhibits the production of high-quality summer tea. To explore the effect of shading on reducing the temperature, we recorded the DMT of the air and soil. The four shading treatments had significant effects on reducing the DMT of air, especially T4, where the double-layer shading nets were 50 cm higher than the plant canopy (Figure 5a–c). In addition, there were no significant differences in the DMT of soil among different shading treatments (Figure 5c). A previous study found that caffeine content was closely correlated with temperature, and our data showed that caffeine content in summer tea was negatively related to the DMT of air (Figure 3e) [2]. However, caffeine accumulation was inhibited by high temperatures in the spring [26,27]. Combined with the previous experimental results, the caffeine content in tea was increased by sub-high temperatures when the weather was suitable for tea plants and decreased by lower temperatures when the weather was hot. This problem needs in-depth study to make it clear.

High air temperatures increase the transpiration and evaporation of the tea garden, resulting in a decrease in the soil water content. In this experiment, the fluctuation of DSWC in shading treatments was not as severe as that in control, and the DSWC of four shading treatments is quite different (Figure 6a,b). Compared to single-layer-net shadings, double-layer-net shadings had a lower value of DSWC (Figure 6c,d). Liu et al. [28] thought that shed-frame covering was more stable in the microclimate of tea gardens than direct covering. However, our results showed that the shed-frame shadings had higher values of the DSWC than direct covering shadings, but were not more stable (Figure 6c,d). In addition, the DSWC had a negative correlation with the content of total free amino acids (Figure 3c). Amino acid metabolism is one of the top five metabolic categories that respond to drought stress [27]. Lin et al. [29] found that deficit irrigation would cause moderate water stress to tea plants, and the freshness of tea leaves is similar to that under full irrigation, but the content of theanine is increased. Therefore, T2 had the lowest DSWC but the highest content of total free amino acids (Figure 3c and Figure 6).

Shading not only affects the temperature and soil water content of the tea garden but also changes the light environment of tea plants. The shading rate was almost 90% in T1 and T3 and was almost 99% in T2 and T4 (Table 1). There is no doubt that sunlight provides energy to promote carbon metabolism, including TP. Therefore, the light transmittance was positively correlated with the content of TP (Figure 3b, Table 1). Similarly, catechin derivatives, about 70–80% of TP in fresh tea leaves, decreased when the shading level increased [11,30]. At the same time, the total AA was positively correlated with the shading rate, and the AA content in the treatment with a 99% shading rate was the highest (Figure 3c, Table 1). Light-sensitive tea cultivars such as ‘Huangjinya’, ‘Yu-Jin-Xiang’, and ‘Baijiguan’ have higher AA content than green tea cultivars [18,20,31,32]. When plants were nurtured in the dark, most amino acids, including L-phenylalanine, the key precursor of volatile compounds, were significantly enhanced in *C. sinensis* var. Yabukita [33]. Furthermore, studies revealed that two chloroplast proteases, ATP-dependent Clp protease proteolytic subunit 3 and protease Do-like 2, were up-accumulated in dark-treated leaves and contributed to amino acid accumulation in dark-treated leaves [34]. These indicated that light is a key factor in the metabolism of amino acids.

Light quality, just different wavelengths of light, plays roles in plant growth and resistance. Light within 400–700 nm, including R light and B light, is most effective for photosynthesis [35]. Previous studies found that R light and B light had effects on the quality of tea, including flavonoid metabolism and amino acid accumulation [29,36,37]. Here, although the intensity of light decreased, there was no difference in the percentages of R and B among the four shading treatments (Figure 7a,b, Table 1). On the contrary, shading increased the percentage of FR light, especially in T2 and T4 (Figure 7c). For this reason, the ratio of R light to FR light was sharply down in T2 and T4 (Figure 7d). In recent years, studies have found that not only visible light but also FR light could mediate plant growth [38,39,40,41]. Tatsoi is a variety of *Brassica rapa*, and supplemental FR light decreased their growth and amino acid content [42]. Opposed to tatsoi, bread wheat and lettuce accumulated amino acids such as glutamic acid, glutamine, and aspartic acid under FR light [43,44]. Similarly, our results showed that shading-promoted FR light may be positively related to the content of theanine in tea, and this was worth exploring (Figure 3c, Figure 4, and Figure 7c).

## 4. Materials and Methods

### 4.1. Plant Materials, Treatments, and Sample Collection

Fifteen-year-old *Camellia sinensis* cv. ‘Longjing 43’ tea cultivar grown in the tea garden of the Tea Research Institute, Chinese Academy of Agricultural Sciences, Hangzhou, Zhejiang province, China (longitude 120°100′ E and latitude 30°140′ N, 16 m above sea level) was studied in this experiment. The experiment was conducted in the summer for 14 days (from 27 June 2021 to 10 July 2021). Black polyethylene nets (shade level is about 90% per layer of net; see Table 1) were used for shading. The treatments were as follows: ‘Control’ without shading, ‘T1’ with a one-layer shading net on the canopy of tea plants, T2 with double-layer shading nets on the canopy of tea plants, T3 with a one-layer shading net 50 cm above the canopy of tea plants, and T4 with a double-layer shading net 50 cm above the canopy of tea plants. In this study, three replicates were used for each treatment, and each replicate used a 50-m-long tea row and about 100 tea plants per row.

Microenvironmental factors, such as temperature, soil water content, and light, were tested during the experiment. After 14 days of treatment, all the fresh leaf samples with one bud and two leaves from the experimental tea row were collected to measure the yield and quality of the tea. When calculating tea yield, use the formula Y (kg∙ha^−1^) = W/(l × w) × 10^4^. In this formula, W represents the weight of fresh tea leaves (kg), and l and w represent the length of the row (50 m) and the width of the row (1.5 m), respectively. For quality examination, tea samples were dried and ground.

### 4.2. Gas Exchange and Chlorophyll Measurement

The net photosynthetic rate (P_N_), transpiration rate (Tr), and conductance to H_2_O (Gs) were measured on the first and second mature leaves below one bud and two leaves by using an open-flow infrared gas analyzer adapted with light and temperature control systems (Li-6400XT, LI-COR, Lincoln, NE, USA). The CO_2_ concentration and photosynthetic photon flux density (PPFD) were maintained at 400 mmol∙mol^−1^ and 800 mmol∙m^−2^∙s^−1^, respectively. SPAD is the relative content of chlorophyll, measured by SPAD-502 Plus (Konica, Japan). Gas exchange and the relative amount of chlorophyll measurements were performed within the period from 8:00 am to 10:00 am on 10 July 2021. Three replicates were measured for each treatment.

### 4.3. Nutrient Elements Measurement

The protocol for the phosphorus (P) measurement was described previously [45]. Briefly, plant samples were digested in concentrated sulfuric acid at 150 °C, and 30% hydrogen peroxide was used to promote the redox reactions. The homogenates were filtrated and diluted with distilled water. The pH value was adjusted to 7–8. P measurements were conducted according to Delhaize and Randall [46].

For measuring other nutrient elements, the plant samples were powdered and weighed, then ashed at 500 °C for 10 h. The ash was digested with 30% HNO_3_ and diluted to a final volume of 25 mL using deionized water. The concentrations of nutrient elements were measured by flame atomic absorption spectroscopy (FAAS, Agilent, Santa Clara, CA, USA).

### 4.4. Tea Quality Measurement

The methods for measuring tea quality were described previously [47]. To assess the quality of tea leaves as influenced by shading treatment, grind the leaves into tea powder and extract the supernatant of the tea infusion. Three biological replicates were measured in each treatment.

For every replicate, accurately 1.000 g of tea powder for one replicate was added in a 50 mL conical flask and mixed with 45 mL ultrapure water. The mixture was boiled in hot water for 45 min (shaking once every 10 min). The hot supernatant was filtered, and the residue was washed with hot ultrapure water 4–5 times. After cooling, the filtrate was transferred into a 50 mL centrifuge tube and filled with ultrapure water.

The contents of tea polyphenols (TP), total amino acids (AA), and caffeine were determined according to GB/T 8313—2018, GB/T 8314—2013, and GB/T 8312—2013, respectively. The ultraviolet spectrophotometer (UV-2550, Shimadzu, Kyoto, Japan) was used for measurement. The wavelengths used for measuring TP, AA, and caffeine were 540 nm, 570 nm, and 274 nm, respectively.

Individual AA was measured by an automatic AA analyzer (L-8900, Hitachi, Tokyo, Japan). Five milliliters of tea extracts were added to 5 mL of sulfosalicylic acid, and the mixture was centrifuged at 13,000× *g* for 5 min to facilitate the reaction. The mixture was filtered through a 0.22 μm nylon filter membrane and run in the AA analyzer.

### 4.5. Temperature and Soil Water Content Measurement

Temperature and soil water content were detected by the sensors, which were connected to an automatic record instrument (L95, Luge, Hangzhou, China). Temperature sensors were installed on the canopy of tea plants and in the soil for temperature. Humidity sensors were installed in the soil of tea plants to measure soil water content. The instruments automatically detected and recorded temperature and humidity every hour for 14 days (27 June 2021 to 10 July 2021).

### 4.6. Light Spectrum Measurement

The light environment (350–800 nm) on the canopy of tea plants was measured by the plant lighting analyzer (PLA-30, Everfine, Hangzhou, China) at 10 o’clock every morning for 7 days (July 3rd to July 10th, 2021). The measured data includes PPFD (μmol·m^−2^·s^−1^, 400–700 nm), PPFDf (μmol·m^−2^·s^−1^, 700–800 nm), total photometric irradiance (W·m^−2^, 350–800 nm), as well as specific photometric irradiance of blue light (B, W·m^−2^, 400–500 nm), red light (R, W·m^−2^, 600–700 nm), and far-red light (FR, W·m^−2^, 700–800 nm). The percentages of B, R, and FR were calculated by the ratio of specific photometric irradiance to total photometric irradiance.

### 4.7. Statistical Analysis

For the measurements, at least three biological replicates were used for each treatment. Data were statistically analyzed by one-way ANOVA using the SPSS26 statistical package. Shared letters indicate no statistically significant difference in the means (*p* > 0.05) after using the *LSD* test. The data visualization was created by GraphPad Prism 9.

## 5. Conclusions

As mentioned above, shading reduced plants’ photosynthesis and tea yield but improved the tea quality in summer, and the double-layer-net shadings had better effects on the quality of tea leaves. Furthermore, T4, which used double-layer shading nets 50 cm above the canopy of tea plants, exhibited greater stability in air temperature and soil water content, as well as an increased level of FR irradiation. Due to comprehensive improvements in environmental factors, T4 was better for the accumulation of umami and sweet amino acids in tea leaves. These results provide bases for the efficient summer shading of tea plants and investigating the role of the microenvironment in regulating tea quality.

## Figures and Tables

**Table 1:** The PPFD and light transmittance on the canopy of tea plants as influenced by different shading treatments.

Group Name	PPFD (μmol·m^−2^·s^−1^)							Light Transmittance (%)
	D1	D2	D3	D4	D5	D6	D7	
Control	512.83	1110.40	761.27	1186.30	513.85	230.24	333.28	/
T1	50.15	16.47	67.05	70.35	65.87	27.66	32.19	8.64 ± 3.87 b ^1^
T2	7.75	2.23	10.32	11.45	8.89	3.87	5.99	1.32 ± 0.57 c ^1^
T3	77.82	62.19	90.99	105.32	68.36	30.61	50.99	11.93 ± 3.54 a ^1^
T4	11.81	3.75	19.50	16.96	12.11	6.41	7.00	1.98 ± 0.84 c ^1^

^1^ Light transmittance (%) is the average of seven replicates (±SD) from the later seven days of treatment (D1–D7, from 4–10 July 2021). Shared letters indicate no statistically significant difference in the means (*p* > 0.05) according to the *LSD* test.

## Data Availability

The data presented in this study are available on request from the corresponding author. The data are not publicly available due to privacy.
